# An Improved Trilateration Positioning Algorithm with Anchor Node Combination and K-Means Clustering

**DOI:** 10.3390/s22166085

**Published:** 2022-08-15

**Authors:** Qinghua Luo, Kexin Yang, Xiaozhen Yan, Jianfeng Li, Chenxu Wang, Zhiquan Zhou

**Affiliations:** 1 School of Information Science and Engineering, Harbin Institute of Technology, Weihai 264209, China; 2 Shandong Institute of Shipbuilding Technology, Ltd., Weihai 264209, China; 3 Shandong New Beiyang Information Technology Co., Ltd., Weihai 264209, China

**Keywords:** trilateration, wireless sensor network, K-Means, Received Signal Strength Indication, localization

## Abstract

As a classic positioning algorithm with a simple principle and low computational complexity, the trilateration positioning algorithm utilizes the coordinates of three anchor nodes to determine the position of an unknown node, which is widely applied in various positioning scenes. However, due to the environmental noise, environmental interference, the distance estimation error, the uncertainty of anchor nodes’ coordinates, and other negative factors, the positioning error increases significantly. For this problem, we propose a new trilateration algorithm based on the combination and K-Means clustering to effectively remove the positioning results with significant errors in this paper, which makes full use of the position and distance information of the anchor nodes in the area. In this method, after analyzing the factors affecting the optimization of the trilateration and selecting optimal parameters, we carry out experiments to verify the effectiveness and feasibility of the proposed algorithm. We also compare the positioning accuracy and positioning efficiency of the proposed algorithm with those of other algorithms in different environments. According to the comparison of the least-squares method, the maximum likelihood method, the classical trilateration and the proposed trilateration, the results of the experiments show that the proposed trilateration algorithm performs well in the positioning accuracy and efficiency in both light-of-sight (LOS) and non-light-of-sight (NLOS) environments. Then, we test our approach in three realistic environments, i.e., indoor, outdoor and hall. The experimental results show that when there are few available anchor nodes, the proposed localization method reduces the mean distance error compared with the classical trilateration, the least-squares method, and the maximum likelihood.

## 1. Introduction

With the rapid development of the Internet and information technology, human demands are not only communicating with the computer but also communicating with everything, which led to the birth of the Internet of Things (IoT). Wireless Sensor Networks (WSN) are the primary department of the IoT. A WSN is comprised of a large number of sensors, which are used to sense and identify the information of the surrounding environment [1]. A WSN can be used in various applications such as smart agriculture, defense, industry, and health care. In a WSN, the most basic information is the location information of the object. Algorithms are very important for the whole process of localization. The most commonly used positioning system is the Global Positioning System (GPS), but the signal strength of the satellites can be influenced by the buildings and other environments, which is not applicable in an indoor location. Moreover, installing GPS modules on each object will greatly increase the cost, which is not economically feasible. Therefore, wireless sensor networks have gained wide attention in indoor or obscured environments [2].

In recent years, advanced optimization algorithms have been developed rapidly and are widely used in many fields. In a multi-objective optimization problem, a multi-objective problem with different characteristics can be optimized by using machine learning [3]. An angle-based selection strategy and a displacement-based density estimation strategy [4] can be used to select the environment to purify the population. A metric-based evolutionary algorithm is used to prevent the population from being trapped in local areas. In scheduling problems [5], metaheuristic algorithms are often used to solve practical size problems, and evolutionary algorithms can also be used to solve some high-complexity problems. In the differentiation of meningitis, machine learning algorithms such as genetic programming and decision trees [6] are used to correctly differentiate the differentiation of induced meningitis. For the classification of data, other evolutionary algorithms such as genetic algorithm (GA), particle swarm optimization (PSO), differential evolution (DE), and gray wolf optimization (GWO) have been used [7,8,9].

WSN localization methods and algorithms can be classified by different characteristics. The algorithm can be divided into range-free and range-based localization methods. In range-free methods, we utilize the number of hops among nodes and the connectivity of the networks to estimate the position of unknown nodes via specific algorithms. Range-free localization methods have the advantages of simple calculation and low requirement of hardware. For instance, the distance vector-hop (DV-Hop) [10] uses distance vector routing to calculate the average number of hops in the network, which calculates the nodes’ location. APIT (Approximate Point In Triangulation Test) [11] uses the PIT (Point In Triangulation) theory to calculate the nodes’ position by exchanging information through the connectivity of the network. The centroid [12] algorithm uses closed polygons formed by neighboring nodes of the unknown nodes for localization. The core of the range-based localization algorithm is the distance measurement or the angle measurement, which requires hardware support but at the same time achieves high localization accuracy. The instruments are necessary for range-based localization, which are utilized to get the range or angle information. Then, the information is used to calculate the unknown nodes’ position according to specific methods. Furthermore, Time of Arrival (TOA) [13], Time Difference of Arrival (TDOA) [14], Angle of Arrival (AOA) [15], and Received Signal Strength Indication (RSSI) [16] are four common measurement methods. The advantages and disadvantages of the methods are listed in Table 1.

Among these methods, RSSI is widely applied in a WSN because of its low cost and low computational complexity. After obtaining information such as distance or angle, the coordinates of unknown nodes can be obtained by performing operations using geometric or algebraic relationships between them. Trilateration uses the three neighboring nodes of the unknown node and their distances to the unknown node to calculate the coordinates of the unknown node. The trilateration algorithm is easy to understand and has low computational complexity, but in the process of actual measurement, there are often large measurement errors leading to the situation that the three circles do not intersect. Triangulation uses the angle relationship between the unknown node and the neighboring nodes to calculate the coordinates of the unknown node, but the hardware conditions required to measure the angle are relatively harsh, which leads to the application being relatively small. There are also the maximum likelihood method [17] and the least-squares method [18] that use algebraic relations to find the coordinates of unknown nodes. These methods can minimize the square of the error and find the best functional match of the data, and are simple and accurate when there are enough anchor nodes. However, these methods are linear estimation and have defaulted to a linear relationship, so there is subjectivity. Moreover, in wireless sensor networks, reducing the number of beacon nodes in the network can effectively reduce the cost of the network during the deployment phase and the operation phase [19,20,21]. However, when there are few anchor nodes, the positioning accuracy of the least-squares method and the maximum likelihood method will be greatly reduced.

Trilateration is a classic range-based localization method, which has the advantages of low cost and low computational complexity. So, it is suitable for plenty of applications. Nevertheless, many factors limited the accuracy of the trilateration method in practical application. The main factors affecting the positioning accuracy of the trilateration method are as follows [22]:

- Uncertainty: As a result of the NLOS, the multi-path fading channel (MPF) and the Near-Far Effect (NFE), there are different levels of uncertainties during the measurement processing, which may cause the phenomenon that the three circles intersect on a specific area, or do not intersect on a point. The positioning error will be very significant.
- Heterogeneity: The information of three or even more anchor nodes is required to achieve a wider localization range of coverage in most cases, which leads to the possibility of different results for positioning calculation.
- Error propagation: To improve the positioning ability, we always treat the located nodes as secondary anchor nodes. As there are different degrees of error in the secondary anchor nodes’ localization, error propagation and accumulation will occur when locating other unknown nodes with the secondary anchor nodes.

To improve the positioning performance and obtain more accurate positioning results, we propose an improved trilateration localization algorithm with the combination and K-Means [23] clustering strategy in this paper. We first combine the anchor nodes into multiple trilateration positioning groups. Then, we determine the positioning result with high quality through K-Means cluster algorithms. Finally, we gain a more accurate positing result of the unknown node.

In this paper, we make the following contributions:

- To make full use of the information from the anchor nodes, we propose a novel trilateration method based on the combination and K-Means clustering. The combination is used to generate, which are clustered by the K-Means cluster. We can obtain an accurate position of the unknown nodes.
- We determine the factors influencing the accuracy of the proposed method through tests under different groups of combination and clustering.
- To evaluate the performance comprehensively, we apply the proposed method in LOS and NLOS environments. Additionally, the simulation results show that the proposed method can effectively improve the positioning accuracy.
- In order to verify the feasibility in the real environment, we conduct experiments in three environments: indoor, outdoor and hall. The performance of this method is better than other methods when there are few available anchor nodes.

The rest of this paper is organized as follows: Section 2 introduces the related works about the trilateration method. In Section 3, we introduce the principle of the proposed positioning method. We conduct simulations and demonstrate the results in Section 4. In Section 5, we setup real localization environment and validate the method. In Section 6, we conclude and suggest further research directions.

## 2. Related Works

In recent years, many positioning technologies have been applied in real life and have a wide and important application in several aspects. Since the trilateration localization algorithm requires only the distance between the nodes, it is suitable for research applications where the school bus and each student are constantly moving during the journey. By using Bluetooth Low-Energy Beacon and IoT technology, the location of the children can be timely sent to the driver [24]. Beacons and smartphones can be used to analyze the visitors’ locations, and deliver relevant cultural content to visitors near artworks. The time visitors spend near different artworks can be captured in order to make recommendations for future museum settings [25]. Location technology also plays a very important role in inventory management, helping employees in the warehouse to identify and track goods [26].

To solve the problems mentioned in Section 1, many scholars presented various novel methods based on the trilateration for the current problems in practical applications.

The authors of [2,27] utilized a trilateration algorithm to get the nearest access point and then processed the data using the Extended Kalman Filter (EKF) and the Kalman Filter (KF), respectively. The authors of [28] proposed a novel NLOS-aware localization algorithm, namely Enhanced Fingerprinting-aided RSS-TL (EFP-RSS-TL). It used the RSS fingerprint database, which recorded the line-of-sight (LOS) light power ratios beforehand to eliminate the NLOS impact, which could improve the localization accuracy in the NLOS environment. The authors of [29] proposed a novel least-squares curve fitting (LSCF) method in RSSI-based localization to decrease the dependence on the anchor node’s position and RSSI measurements. Then, the trilateration was used to calculate the localization of the unknown node. Based on the trilateration and the selection of the anchor nodes, the authors of [30] proposed an adaptive range-based localization (ARBL) algorithm. The algorithm can find the best combination of the anchor nodes at a given time. The authors of [31] applied an Artificial Neural Network (ANN) to RSSI measurements from three Bluetooth Low-Energy (BLE) Beacons to solve the problem of the fluctuations caused by human body shadowing. The distances obtained can be applied to the trilateration. To overcome the issues of no or multiple intersect points in trilateration, the authors of [32] proposed a novel Weighted Concentric Circle Generation (WCCG) method to increase the possibility of finding intersect points. Then, mean shift clustering is used to determine the node’s localization. The authors of [33] listed and classified all possible cases of errors in trilateration and proposes a separate estimation algorithm. The authors of [34] proposed a new geometric method that covered all the cases mentioned in [33] and avoided case partitioning. A new trilateration method based on extreme value theory was proposed in [35]. A non-linear error function was constructed according to the distance and the position of the anchor nodes. The coordinate of the unknown node is the value that minimizes the error function. The authors of [36] proposed an improved trilateration localization algorithm that calculates the uncertainty in the ranging process of all anchor nodes, selects the anchor node with the smallest uncertainty propagation among the anchor nodes using the single-scan sliding window in the optimization algorithm, and then obtains accurate localization results based on the selected three anchor nodes by the least-squares criterion. This method has very good scalability and can be applied not only to trilateration the measurement method, but also to least squares and great likelihood methods to obtain high localization accuracy and acceptable localization efficiency. The authors of [37] proposed an improved algorithm based on RSSI ranging, which can improve the localization accuracy by increasing the anchor nodes’ density. The unknown node which was located can be upgraded into a new anchor node. However, in the process of upgrading, there is the problem of error accumulation, i.e., the newly upgraded anchor node already has uncertainty in its position, and this error will be transferred to the localization process of the new unknown node. The authors of [38] combined the fingerprint algorithm with trilateration to provide the additional distance for localization, especially when the number of the anchor nodes is not sufficient. It is suitable for improving the accuracy of Ultra-Wide Bandwidth (UWB) localization systems in a complex indoor environment with a limited number of anchor nodes. However, its prerequisite is a fingerprint database with sufficient density.

The method of clustering is often applied in many localization algorithms. The cluster analysis divides data into groups or clusters based on certain parameters so that data with similar parameters are together in one cluster [39]. The clusters with small numbers will be considered as falsely detected target positions. These positions will be eliminated in order to improving the localization accuracy. An improved method is proposed in [40] to use Ultra-Wide Bandwith (UWB) to positioning by adding cluster analysis processing and using K-Means clustering to eliminate incorrect target detection that occurs in conventional processing. In [41], a localization method of WiFi signal sources is proposed for detecting and tracking fake access points (AP) scenarios. The coordinates of multiple groups of centroids are initially calculated using the triangular centroid positioning method, and then the results are processed using the K-Means clustering algorithm. Additionally, appropriate weight values are selected according to the size of each cluster. The method has high positioning accuracy and robustness. However, the method has the disadvantage of high calculation volume. In [42], the indoor joint positioning algorithm combines the Kalman Filtering algorithm and the clustering algorithm, determines the initial value of the signal through K-Means, then uses Gaussian Mixture Model (GMM) algorithm to cluster the fingerprint database, and corrects the estimated position to be measured by the Kalman Filtering algorithm in the online stage. Inspired by the above literature, we propose an improved method in this paper: an improved trilateration positioning algorithm with anchor node combination and K-Means clustering.

## 3. The Improved Trilateration Method

**Figure 1:**
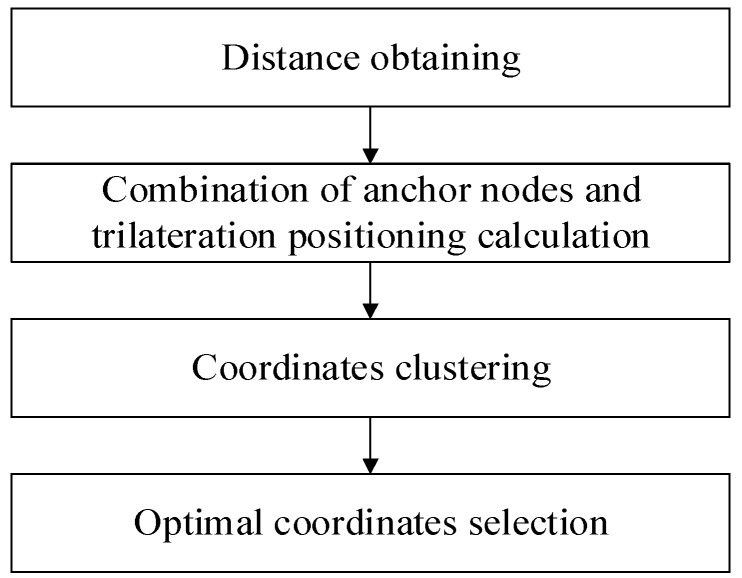
The flowchart of the improved trilateration based on the combination and clustering strategy.

We illustrate the framework of the improved trilateration positioning algorithm based on the combination of anchor nodes and K-Means clustering in Figure 1. It comprises the following sub-modules: distances obtaining, combination of anchor nodes and trilateration positioning calculation, coordinate clustering, and optimal coordinate selection.

The implementation steps of each part are as follows:

- **Distance obtaining**: We estimate the distances between the unknown nodes and all anchor nodes through RSSI. We obtain the distances for trilateration positioning.
- **Combination of anchor nodes and trilateration positioning calculation**: To take advantage of all the anchor nodes, we combine the anchor nodes into many groups according to the combination. The anchor nodes are grouped in order to obtain the estimated coordinates, which are the samples in the cluster.
- **Coordinate clustering**: K-Means clustering is used to group the samples into *k* clusters.
- **Optimal coordinate selection**: In the sample set, the centroids of the clusters will drift to the direction of the samples with large errors, which leads to the decrease in the number of samples in this cluster. Hence, we consider the final estimated position as the centroid of the cluster, which has the largest number of samples.

### 3.1. Distance Obtaining

In this section, we utilize RSSI to obtain the distances between the unknown nodes and anchor nodes. RSSI is a measurement method using the strength of the received signal to estimate the distance between the nodes. The strength of the transmitted signal and the strength of the signal received by the receiving node are known, then the difference between the two strengths is the propagation loss. The distance can be obtained by substituting the loss value into the corresponding model. A conventional definition of RSSI to the distance *d* is given as:

(1) RSSI=−n×10×lg(d)−A

where *n* is a propagation constant; *A* is the reference RSSI in dBm, which depends on the default channel attenuation in the space. With the referential RSSI model, an extensive formula is given in [43,44] to define the RSSI at *d* distance to the transmitter:

(2) $${\mathrm{RSSI}}_{\mathrm{dB}}(d)={\mathrm{RSSI}}_{\mathrm{dB}}(d_{0})-n\times 10\times \mathrm{lg}(\frac{d}{d_{0}})$$

where *d*_0_ denotes the distance from referenced point to the common transmitter, and RSSI_dB_(*d*_0_) is the corresponding RSSI measured at *d*_0_ distance to the transmitter. *n* is the path loss exponent.

To simulate the uncertainty in the actual positioning environment, the real RSSI value is derived from the real distance value in the positioning scene, and then different types of noise are superimposed to simulate the wireless signal line-of-sight transmission and wireless signal non-line-of-sight transmission.

### 3.2. Combination of Anchor Nodes and Trilateration Positioning Calculation

#### 3.2.1. Combination of Anchor Nodes

In many positioning environments, there are some abundant anchor nodes with different quality distances. So, we can make full use of these anchor nodes. We first combine these anchor nodes into different groups. In the process of the node’s positioning, the unknown node will receive information from multiple nodes and get multiple distances, while the simple trilateration only utilizes the distance information of three anchor nodes which causes the waste of information. Therefore, the proposed method utilizes the combination of anchor nodes when the distance information of multiple anchor nodes is received. Combinations can take all combinations of *m* (*m* ≤ *n*) elements from *n* different elements [45]. Suppose there are m anchor nodes in the communication scope of an unknown node. According to permutation and combination law, we just select three nodes from the m anchor nodes into a trilateration calculation group. So, the number N of anchor node groups can be calculated shown as in Equation (3).

(3) $$N=C_{m}^{3}=\frac{m(m-1)(m-2)}{3!}$$

Then, we obtain N groups anchor nodes {((*x_A_*_1_, *y_A_*_1_), (*x_B_*_1_, *y_B_*_1_), (*x_C_*_1_, *y_C_*_1_)), ((*x_A_*_2_, *y_A_*_2_), (*x_B_*_2_, *y_B_*_2_), (*x_C_*_2_, *y_C_*_2_)), *…*, ((*x_Ai_*, *y_Ai_*), (*x_Bi_*, *y_Bi_*), (*x_Ci_*, *y_Ci_*)),…((*x_AN_*, *y_AN_*), (*x_BN_*, *y_BN_*), (*x_CN_*, *y_CN_*))} with corresponding distance{(*d_A_*_1_, *d_B_*_1_, *d_C_*_1_), (*d_A_*_2_, *d_B_*_2_, *d_C_*_2_), *…*, (*d_Ai_*, *d_Bi_*, *d_Ci_*_1_), *…*, (*d_AN_*, *d_BN_*, *d_CN_*)}. Here, *i* is the serial number of anchor nodes, and 1≤i≤N.

In practical processing, due to the processing efficiency requirement, we do not determine all the groups of anchor nodes. We will evaluate the impact of group numbers on the positioning performance and determine the appropriate group number in Section 4.2.1.

#### 3.2.2. Trilateration Positioning Calculation

**Figure 2:**
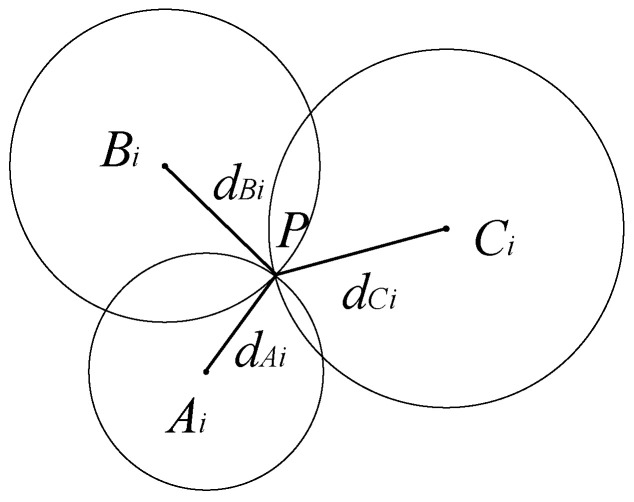
The diagram of trilateration positioning calculation.

Based on the combination of anchor nodes, we gain many different groups. For each group, we will perform trilateration. Assume that *A_i_, B_i_*, and *C_i_* are three anchor nodes in the *i*th group, which are located in (*x_Ai_, y_Ai_*), (*x_Bi_, y_Bi_*), (*x_Ci_, y_Ci_*), and *P* is the target node which is located in (*x, y*). The distances from the target node to three anchor nodes are *d_Ai_, d_Bi_*, *d_Ci_*. An intersection point can be obtained by taking *A_i_, B_i_, C_i_* as the center and *d_Ai_, d_Bi_, d_Ci_* as the radius to make three circles. This intersection point corresponds to the position of the unknown node *P*. The principle is shown in Figure 2.

According to the distances formula between the unknown nodes and anchor nodes. We can obtain the following equations [46,47]:

(4) $$\left\{\begin{array}{l} d_{A}=\sqrt{\left[{\left(x_{A}-x\right)}^{2}+{\left(y_{A}-y\right)}^{2}\right]}\\ d_{B}=\sqrt{\left[{\left(x_{B}-x\right)}^{2}+{\left(y_{B}-y\right)}^{2}\right]}\\ d_{C}=\sqrt{\left[{\left(x_{C}-x\right)}^{2}+{\left(y_{C}-y\right)}^{2}\right]} \end{array}\right.$$

By solving the above equations, the coordinate (*x*, *y*) of the target node *P* can be obtained as:

(5) $$\left[\begin{array}{c} x\\ y \end{array}\right]={\left[\begin{array}{cc} 2x_{A}-2x_{C} & 2y_{A}-2y_{C}\\ 2x_{B}-2x_{C} & 2y_{B}-2y_{C} \end{array}\right]}^{-1}\times \left[\begin{array}{c} x_{A}^{2}-x_{C}^{2}+y_{A}^{2}-y_{C}^{2}+d_{C}^{2}-d_{A}^{2}\\ x_{B}^{2}-x_{C}^{2}+y_{B}^{2}-y_{C}^{2}+d_{C}^{2}-d_{B}^{2} \end{array}\right]$$

In the same way, we perform the trilateration calculation on other groups and get the corresponding coordinates of the unknown node. So, we can gain *N* groups of coordinates of the unknown node: ***X*** = {(*x*_1_, *y*_1_), (*x*_2_, *y*_2_), …, (*x_i_*, *y_i_*), …, (*x_N_*, *y_N_*)}.

### 3.3. Coordinate Clustering

**Figure 3:**
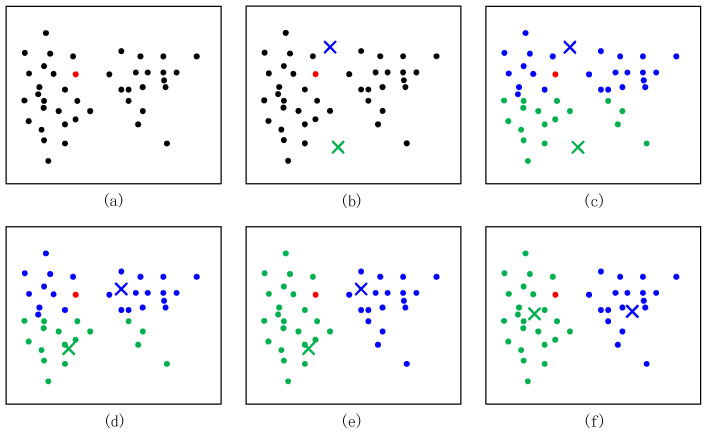
The diagram of the K-Means clustering.

**Figure 4:**
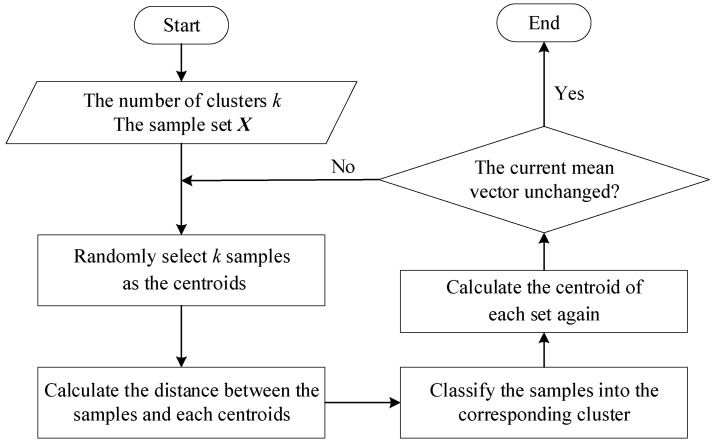
The flowchart of the coordinate clustering.

After anchor node combination and trilateration positioning calculation, we gain *N* coordinates of the unknown node. We utilize the cluster to increase accurate positioning results. Due to the error in distance measurement, there may be no intersecting area of the three circles or other situations, resulting in a large error in the results sometimes. However, the probability of the above situation is small. Therefore, the K-Means clustering can be applied to filter these situations out. K-Means clustering can automatically divide the same elements into closely related clusters. In the proposed trilateration, the computational coordinates of several unknown nodes can be obtained by the combination and trilateration algorithm. If we simply consider the center of mass of these coordinates as the final coordinates of the unknown nodes, it is inevitable that they will be affected by some of these coordinates with large errors, resulting in large deviations in the final results. We hope to remove some of these coordinates with large errors, and these coordinates can be removed using K-Means clustering. The diagram is shown in Figure 3. The flowchart is shown in Figure 4.

The original sample set is expressed in Figure 3a. Assume that *k* = 2. In Figure 3b, we randomly select two points as the centers of the cluster, i.e., the red center and the blue center. Then, we calculate the distance between the samples and each center, and the category of each sample is labeled as the category of the center with the smallest distance from that sample. As shown in Figure 3c, we obtain the result of the first iteration. Then, we calculate the new centers of the cluster and obtain Figure 3d. Figure 3e,f repeat the processes of Figure 3c,d, which is the process that satisfies the samples. Finally, we can obtain two clusters, as shown in Figure 3f.

Assuming that the sample set ***X***= {***X***_1_ = (*x*_1_, *y*_1_), ***X***_2_ = (*x*_2_, *y*_2_), …, ***X***_i_ = (*x_i_, y_i_*), …, ***X****_N_* = (*x_N_, y_N_*)} includes *N* samples. The K-Means cluster divides the sample set ***X*** into *k* disjoint clusters {*C_l_*|*l* = 1, 2, …, *k*}. The cluster label is expressed by *λ_j_*∈{1, 2, …, *k*}, and ***X****_j_*∈$C_{\lambda_{j}}$. The specific K-Means clustering process will be given below Algorithm 1.
**Algorithm 1: Coordinate Clustering based on K-Means**    **Input**: Cluster number, *k*; sample set, ***X*** = {***X***_1_, ***X***_2_, …, ***X_N_***};    **Output**: Cluster center, *C* = {*C*_1_, *C*_2_, …, *C_k_*};    Randomly select *k* samples as the initial centroids {***μ***_1_, ***μ***_2_, …, ***μ**_k_*};    Repeat        $C_{i}=\emptyset ,(1\leq i\leq k)$        for *j* = 1, 2, …, *N* do          Calculate the distance between sample ***X**_j_* and each mean vector $\mu_{i}(1\leq i\leq k)$: $d_{ij}=∥X_{j}-\mu_{i}{∥}_{2}$;          Determined the cluster market of ***X_j_*** according to the nearest mean vector: $\lambda_{i}=\arg\min_{i\in \{1,2,\ldots k\}}d_{ij}$;          Classify the sample ***X**_j_* into the corresponding cluster: $C_{\lambda_{j}}=C_{\lambda_{j}}\cup \{X_{j}\}$;      end for      for *i*=1, 2, …, *N* do          Calculate new mean vector: $\mu_{i}^{\prime }=\frac{1}{\left|C_{i}\right|}\sum_{X\in C_{i}}X$;        if $\mu_{i}^{\prime }\neq \mu_{i}$ then            Update the current mean vector $\mu_{i}$ to $\mu_{i}^{\prime }$;        else            Keep the current mean vector unchanged;        end if      end for Until the current mean vector is not updated

### 3.4. Optimal Coordinate Selection

Based on the output of the coordinate clustering, we select the coordinate optimal from the cluster centers *C* = {*C*_1_, *C*_2_, …, *C_k_*}, 1≤j≤k. Considering the position results with significant error will warp the cluster center during clustering. The member number of the corresponding cluster will be small. So, the quality of coordinate in the cluster with maximum member *M_j_* is more accurate. We select the corresponding cluster center *C_j_* as the positioning result. So, we gain the final position result (*x*, *y*).

(6) $$\left(\begin{array}{c} x\\ y \end{array}\right)=\underset{M_{j}=\max imum}{C_{j}}$$

### 3.5. Algorithm Time Complexity Analysis

From Equation (5), we can see that the time complexity of the trilateration is *O* (1), and the main time spend of the combined trilateration algorithm is the process of multiple node positioning [48]. Assuming that there are *k* (*k* > 3) anchor nodes participate in positioning, the trilateration calculate times *N* is shown in Equation (7) according to Equation (3).

(7) $$N=C_{k}^{3}=\frac{k(k-1)(k-2)}{3!}$$

The time complexity of *N* positioning is:

(8) $$O(C_{k}^{3})=O(\frac{k(k-1)(k-2)}{3!})$$

When *k* is large, the constant terms and the constant coefficients can be omitted. The time complexity of *N* positioning is close to: *O*(*k*^3^). When a smaller number of anchor nodes is confirmed, the time complexity of *N* positioning is much small than *O*(*k*^3^). The time complexity of K-Means clustering is nearly linear, which is *O*(*nkt*), in which *n* is the number of estimated positions, *t* is the number of iterations of the algorithm, and *k is* the number of clusters. In summary, the time complexity of the proposed method is *O(k^3^*).

## 4. Simulation Evaluation

In this section, we conduct simulations to demonstrate the localization accuracy and robustness of the proposed algorithm. Firstly, we setup an appropriate positioning environment, in which all of the following simulations will be accomplished. Then, the simulation analysis for feasibility was carried out. We evaluate the influence of different parameter settings on the algorithm performance. Additionally, we determine the appropriate values of these parameters. Finally, we evaluate the performance of the proposed improved trilateration method comprehensively and compare it with other related positioning methods. The final simulation results illustrate that the improved trilateration method has a better performance.

### 4.1. Setting Up the Simulational Environment

#### 4.1.1. Computer Configuration

The configuration of the evaluation platform is as follows:

CPU: Intel (TM) core i5 8250H CPU@1.60 GHz, 8G RAM, Windows 10 64 bit.
Evaluation environment: Matlab R2018a.

#### 4.1.2. Simulational Environment Setup

**Figure 5:**
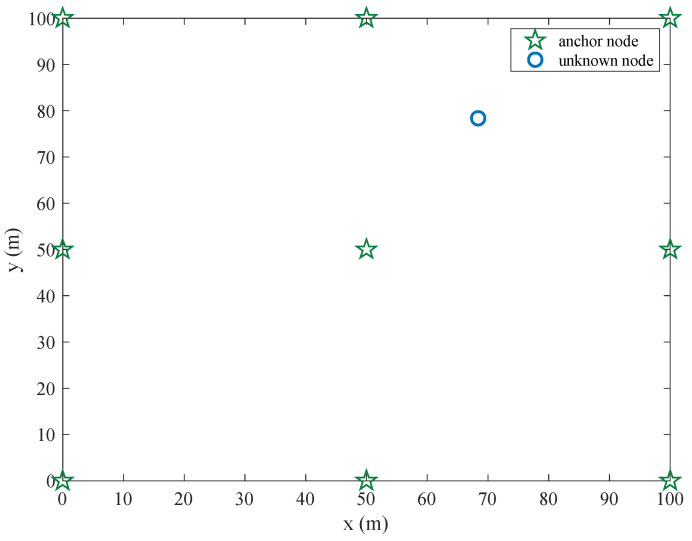
The positioning scenario and setting.

The positioning scenario is set in a 100 (m) × 100 (m) two-dimensional positioning area, as shown in Figure 5, in which 9 anchor nodes are deployed. These 9 anchor nodes are evenly deployed in the localization area, and the unknown node is randomly deployed in the localization area.

#### 4.1.3. Reference Methods

To validate the performance of the proposed method, we select some typical positioning methods as references. The reference methods include the least-squares method, the maximum likelihood method, the random selection trilateration method, and the fixed selection trilateration method. The core principle of the least-squares method and the maximum likelihood method is to form a system of equations that is constructed from the information of the nodes.

#### 4.1.4. Evaluation Parameters

For the performance evaluation, we consider positioning accuracy and positioning efficiency. We evaluate the accuracy in terms of positioning error. Lower positioning error indicates higher accuracy. We evaluate the positioning efficiency in terms of positioning processing time. Short processing time shows high positioning efficiency. The Root Mean Square Error (*RMSE*) can be used as a criterion for analyzing the localization accuracy [35].

(9) $$RMSE=\sqrt{\frac{\sum_{i=1}^{N}\left[{\left({\hat{x}}_{i}-x_{i}\right)}^{2}+{\left({\hat{y}}_{i}-y_{i}\right)}^{2}\right]}{N}}$$

where $\left({\hat{x}}_{i},{\hat{y}}_{i}\right)$ are the estimated coordinates of the node *i*. $\left(x_{i},y_{i}\right)$ are the corresponding true coordinates. *N* is the total number of unknown nodes. In this paper, *N* is set to 1.

### 4.2. Feasibility Simulation Analysis

**Figure 6:**
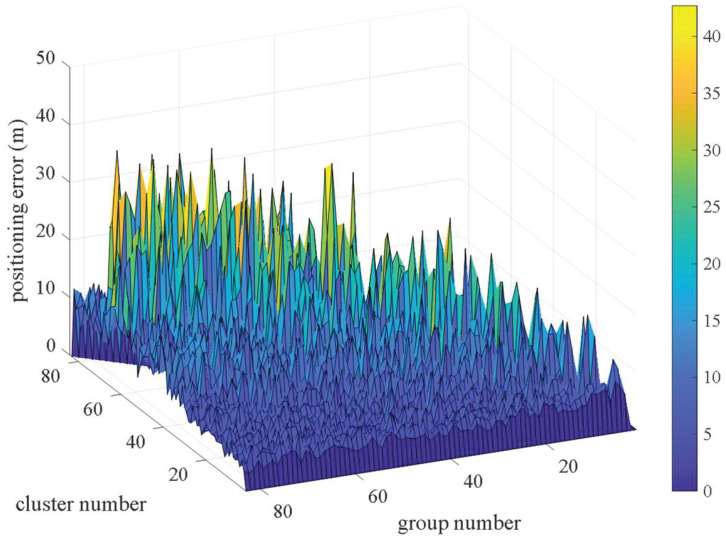
The positioning error changes with the cluster number and group number.

It is obvious that the group number and the cluster number in clustering optimization impact on the performance of this method. In order to observe the influence of different combinations on the positioning results, we set 9 anchor nodes in the simulation environment, which can generate 84 combinations. A total of 100 experiments were performed, and the average positioning error was calculated, and the distribution of the positioning error is shown in Figure 6.

It can be seen from Figure 6 that the positioning error increases with the increment of the cluster number under the situation that the group number keeps constant. By contrast, when the cluster number is fixed, the positioning error changes slowly. The following content will analyze these two factors in detail.

#### 4.2.1. The Influence Analysis of Group Number

**Figure 7:**
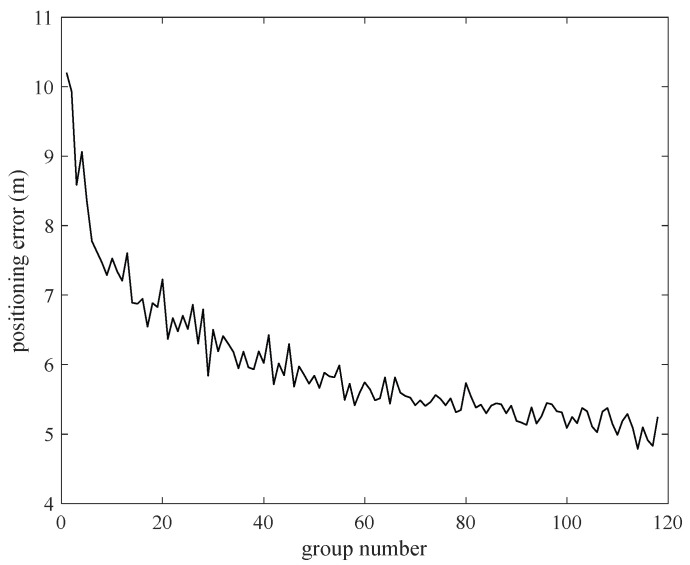
The positioning error changed with different group number.

**Figure 8:**
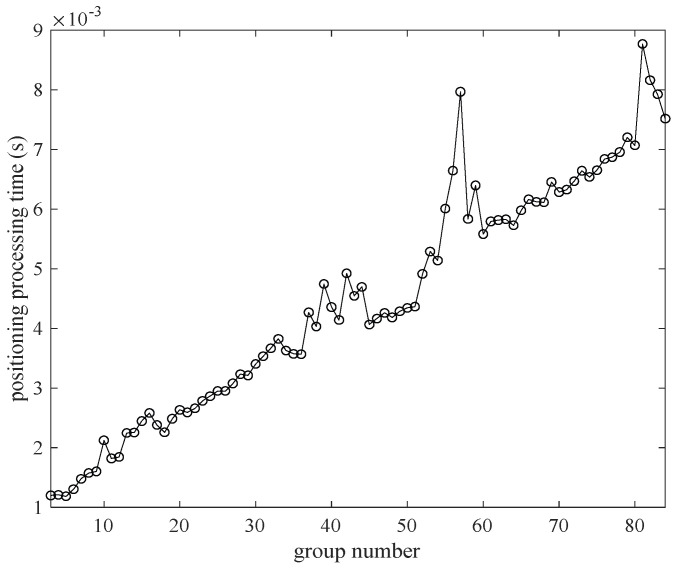
The positioning time changes with different group number.

As can be seen from Figure 6, when the cluster number is fixed, the distribution of the error is similar under different numbers of groups. Thus, we observe the performance of the proposed method with fixed cluster number and different group number. When the cluster number is set to 2, the group number is changed from 3 to 84, and tests are performed 100 times. The average positioning error and the average positioning time are calculated and shown in Figure 7 and Figure 8. It is verified that the average positioning error and the average positioning time of the other cluster number are similar to those when the cluster number is set to 2.

As shown in Figure 7, the average positioning error shows a downward trend as the number of selected groups increases. When the group number reaches the maximum, the positioning error is approximately 33% lower than that of using a group of anchor nodes. This is mainly because each group can obtain an estimated coordinate. The more groups there are, the more coordinates we get. In these circumstances, with the help of clustering, the positioning accuracy of wireless distributed positioning can be improved to a certain extent.

In addition, the processing time of positioning calculation is recorded for the different number of groupings in Figure 8. The processing time is approximately proportional to the number of groupings, and the increasing rate is approximately 0.0038 s per group. This is because a grouping represents once trilateration positioning. The increasing number of the groups means the increasing time of the trilateration. So, the positioning processing time is proportional to the number of groupings.

Considering Figure 7 and Figure 8 comprehensively, if there are too many groups during positioning, the positioning time may be overtime. Sacrificing longer time for smaller positioning accuracy is not economical in the experiment. Considering both positioning error and positioning processing time, we set the group number to 15 in this paper.

#### 4.2.2. The Impact Analysis of Cluster Number

In Figure 6, we can also see that the positioning error increases rapidly when the cluster number is large enough with the same group number. So, we set the group number to 15 to simulate the performance of the method in different numbers of clusters, and 100 times simulation was carried out to calculate the average positioning error and the average positioning time.

**Figure 9:**
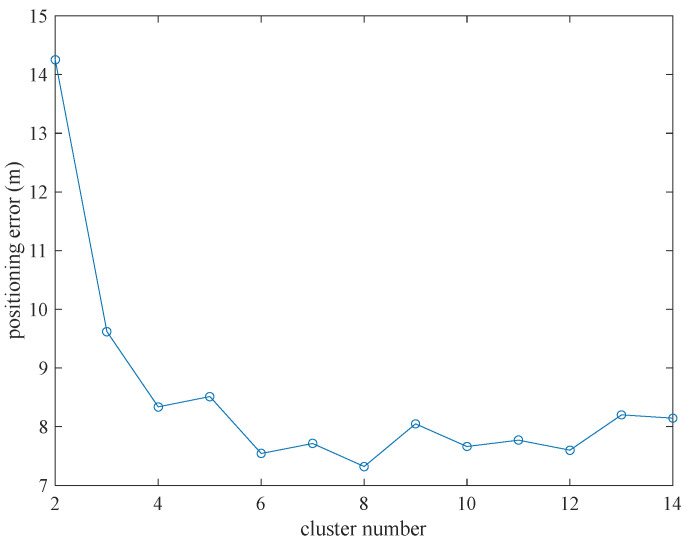
The positioning error changes with different cluster number.

Figure 9 shows the error distribution of positioning in this situation. As shown in Figure 9, when the cluster number is set to 2, the positioning error is the largest and the positioning accuracy is the lowest. The localization error decreases rapidly when the cluster number is greater than 2, and then the positioning error level off. The reason is that when the cluster number is 2, the number of the samples in the cluster is too large, containing many samples with large errors. When the cluster number increases, the number of samples in the clusters decreases, many samples with large errors are divided into other clusters, which causes the decrease in the positioning error. However, there are few samples in the clusters when the cluster number is large enough at the end. Sometimes there is only one sample in the cluster, which leads to an increase in the positioning error.

**Figure 10:**
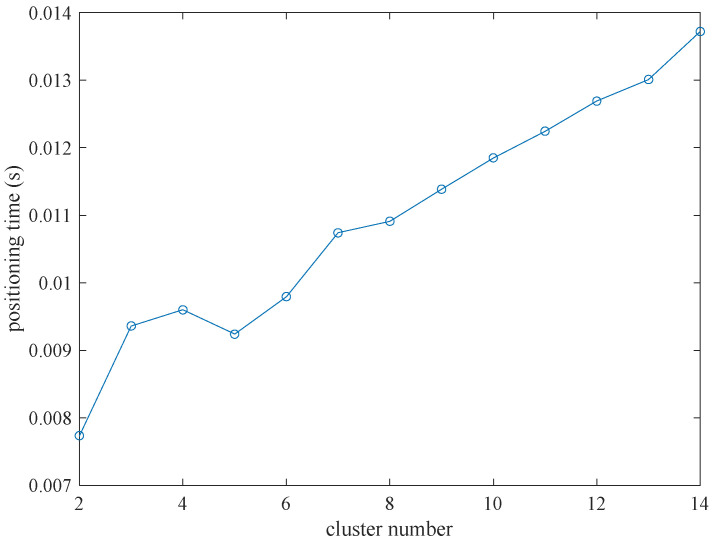
The positioning processing time changes with different cluster number.

We record the positioning processing time with different cluster numbers in Figure 10. Figure 10 shows that with the increase in the cluster number, the time of wireless distributed positioning processing increases proportionally, and the increasing rate is approximately 0.0117 s per cluster. The reason for the increment of positioning time is that with the increase in the cluster number, the calculation amount of centroids increases. Considering both positioning error and positioning processing time comprehensively, two clusters are chosen.

### 4.3. Performance Evaluation

Based on the feasibility simulation analysis, we evaluate the performance of the proposed method in terms of positioning accuracy and positioning processing time. Then, we locate the unknown nodes in the positioning area with algorithms such as the least-squares method, the maximum likelihood method, the classical trilateration and the proposed method in this paper.

- The maximum likelihood method [17]

The coordinate of the unknown node is (*x*, *y*), and the coordinates of the anchor nodes is (*x_i_*, *y_i_*), where 1 < *i* < *n*. The distance from each anchor node to the unknown node is written as *d_i_*. The distance of the *n* equations can be written as:

(10) $$\left\{\begin{array}{c} {\left(x_{1}-x\right)}^{2}+{\left(y_{1}-y\right)}^{2}=d_{1}^{2}\\ {\left(x_{2}-x\right)}^{2}+{\left(y_{2}-y\right)}^{2}=d_{2}^{2}\\ \vdots \\ {\left(x_{n}-x\right)}^{2}+{\left(y_{n}-y\right)}^{2}=d_{n}^{2} \end{array}\right.$$

The above-mentioned equations are dimensionally processed to obtain the binary linear equations of *n* − 1 equations, which are expressed as:

(11) $$\left\{\begin{array}{c} 2\left(x_{1}-x_{n}\right)x+2\left(y_{1}-y_{n}\right)y=x_{1}^{2}+y_{1}^{2}-\left(x_{n}^{2}+y_{n}^{2}\right)+d_{n}^{2}-d_{1}^{2}\\ 2\left(x_{2}-x_{n}\right)x+2\left(y_{2}-y_{n}\right)y=x_{2}^{2}+y_{2}^{2}-\left(x_{n}^{2}+y_{n}^{2}\right)+d_{n}^{2}-d_{2}^{2}\\ \vdots \\ 2\left(x_{n-1}-x_{n}\right)x+2\left(y_{n-1}-y_{n}\right)y=x_{n-1}^{2}+y_{n-1}^{2}-\left(x_{n}^{2}+y_{n}^{2}\right)+d_{n}^{2}-d_{n-1}^{2} \end{array}\right.$$

To rewrite the system of equations into a matrix, let:


(12) $$A=\left(\begin{array}{c} \begin{array}{cc} 2\left(x_{1}-x_{n}\right) & 2\left(y_{1}-y_{n}\right) \end{array}\\ \begin{array}{cc} 2\left(x_{2}-x_{n}\right) & 2\left(y_{2}-y_{n}\right) \end{array}\\ \vdots \\ \begin{array}{cc} 2\left(x_{n-1}-x_{n}\right) & 2\left(y_{n-1}-y_{n}\right) \end{array} \end{array}\right),\;b=\left(\begin{array}{c} x_{1}^{2}+y_{1}^{2}-\left(x_{n}^{2}+y_{n}^{2}\right)+d_{n}^{2}-d_{1}^{2}\\ x_{2}^{2}+y_{2}^{2}-\left(x_{n}^{2}+y_{n}^{2}\right)+d_{n}^{2}-d_{2}^{2}\\ \vdots \\ x_{n-1}^{2}+y_{n-1}^{2}-\left(x_{n}^{2}+y_{n}^{2}\right)+d_{n}^{2}-d_{n-1}^{2} \end{array}\right),\;X={\left(x,y\right)}^{T}$$

The solution using the least-squares method is:

(13) $$X_{Is}={\left(A^{T}A\right)}^{-1}A^{T}b={\left(x,y\right)}^{T}$$

- The least-squares method [18]

Subtracting the two adjacent equations in Equation (11) to obtain Equation (14):

(14) $$\left\{\begin{array}{c} 2\left(x_{2}-x_{1}\right)x+2\left(y_{2}-y_{1}\right)y=x_{2}^{2}+y_{2}^{2}-\left(x_{1}^{2}+y_{1}^{2}\right)+d_{1}^{2}-d_{2}^{2}\\ 2\left(x_{3}-x_{2}\right)x+2\left(y_{3}-y_{2}\right)y=x_{3}^{2}+y_{3}^{2}-\left(x_{2}^{2}+y_{2}^{2}\right)+d_{2}^{2}-d_{3}^{2}\\ \vdots \\ 2\left(x_{n}-x_{n-1}\right)x+2\left(y_{n}-y_{n-1}\right)y=x_{n}^{2}+y_{n}^{2}-\left(x_{n-1}^{2}+y_{n-1}^{2}\right)+d_{n-1}^{2}-d_{n}^{2} \end{array}\right.$$

To rewrite the system of equations into a matrix, let:

(15) $$A=\left(\begin{array}{c} \begin{array}{cc} 2\left(x_{2}-x_{1}\right) & 2\left(y_{2}-y_{1}\right) \end{array}\\ \begin{array}{cc} 2\left(x_{3}-x_{2}\right) & 2\left(y_{3}-y_{2}\right) \end{array}\\ \vdots \\ \begin{array}{cc} 2\left(x_{n}-x_{n-1}\right) & 2\left(y_{n}-y_{n-1}\right) \end{array} \end{array}\right),\;b=\left(\begin{array}{c} x_{2}^{2}+y_{2}^{2}-\left(x_{1}^{2}+y_{1}^{2}\right)+d_{1}^{2}-d_{2}^{2}\\ x_{3}^{2}+y_{3}^{2}-\left(x_{2}^{2}+y_{2}^{2}\right)+d_{2}^{2}-d_{3}^{2}\\ \vdots \\ x_{n}^{2}+y_{n}^{2}-\left(x_{n-1}^{2}+y_{n-1}^{2}\right)+d_{n-1}^{2}-d_{n}^{2} \end{array}\right),\;X={\left(x,y\right)}^{T}$$

Then, the result can be obtained by Equation (13), too.

**Figure 11:**
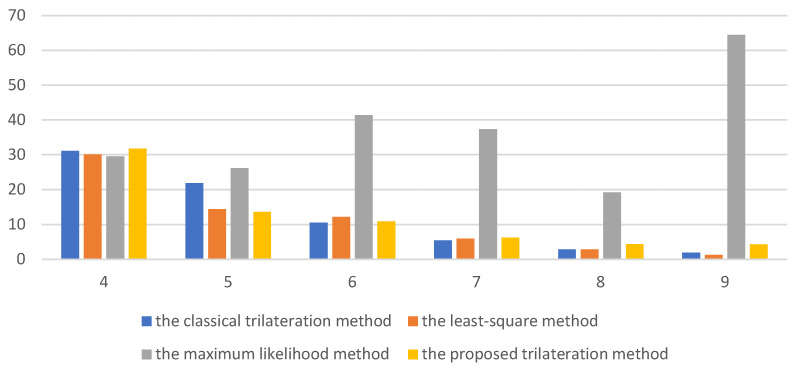
The localization error of different positioning methods.

By comparing the localization errors and processing time, the proposed improved trilateration method is verified to have better performance. In order to observe the performance of the localization algorithm under different numbers of anchor nodes, we change the number of anchor nodes in the localization scene. The number of anchor nodes is increased from 4 to 9 one by one. After performing tests 100 times, the average positioning error is calculated and shown in Figure 11.

From Figure 11, it can be seen that the localization accuracy of the improved trilateration method decreases when the number of the anchor nodes is small. For example, when the number of the anchor nodes is 4, the positioning error of the proposed method in this paper is reduced by 4.95% compared with the least-squares method, by 1.72% compared with the maximum likelihood method, and by 6.98% compared with the classical trilateration method. However, the positioning accuracy of the least-squares method and the maximum likelihood method increases with the increase in the anchor nodes and is similar to that of the improved trilateration method.

It can be seen that the improved trilateration method can achieve the best performance in terms of positioning accuracy when the number of anchor nodes is small. It is because in other methods each anchor node can be utilized only once. However, in the improved trilateration method, the information of the same anchor node and different combinations of anchor nodes produce different results, which interact with each other and can improve the localization accuracy to a certain extent by using the least amount of information.

It can be concluded from Table 2 that when there are less than 9 anchor nodes, the positioning accuracy of the proposed method in this paper is better than that of the least-squares method and the maximum likelihood method. Additionally, it is basically equivalent to that of the least-squares method and the maximum likelihood method when the number of anchor nodes is 9. This is because the improved trilateration method can fully utilize the information of the anchor nodes. In the improved trilateration algorithm, the information of some anchor nodes can be used more than one time. However, in the least-squares method and the maximum likelihood method, the information of each anchor node can only be used once. With the increase in the number of anchor nodes, the information utilization rate of anchor nodes in these three methods gradually tends to be the same. The traditional trilateration has great uncertainty because it only uses one group of anchor nodes. The increase in the number of anchor nodes used for positioning may introduce many estimated coordinates with large errors, resulting in a decrease in the positioning accuracy of the classical trilateration algorithm.

### 4.4. Performance in NLOS

**Figure 12:**
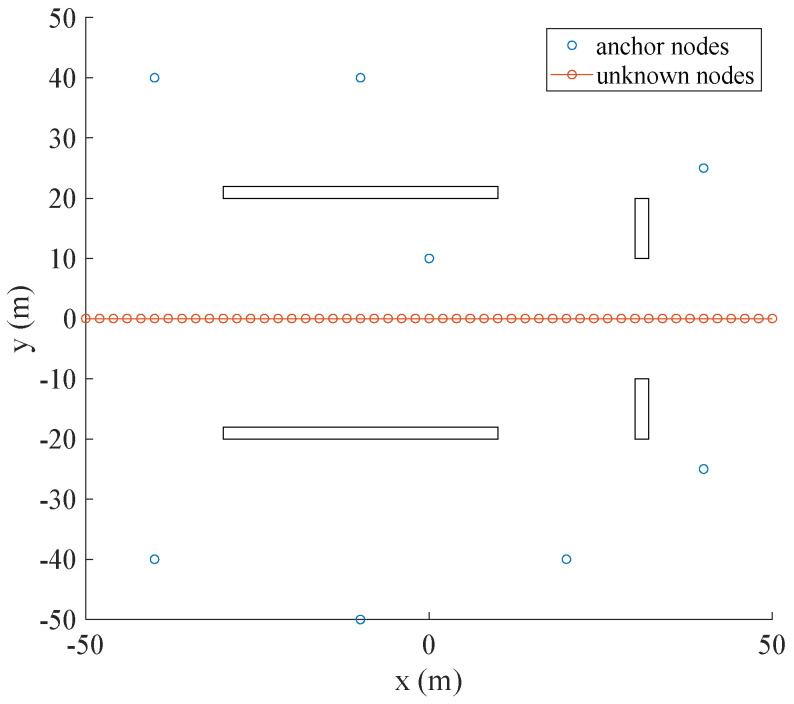
The positioning scenario and settings in the LOS/NLOS mixture environment.

As we know, it is complicated and challenging for the positioning method in a LOS/NLOS mixture environment. We evaluate the performance of the proposed method in a LOS/NLOS mixture environment. We setup a LOS/NLOS mixture environment in the two-dimensional positioning area of 100 (m) × 100 (m), as shown in Figure 12.

There are nine anchor nodes and one unknown node in the positioning scenario above. The unknown node moves from (−50,0) to (50,0) position in a straight line. In the positioning area, four square obstacles are setup to simulate the NLOS environment. In the distance measurement, the LOS environment is created by adding Gauss noise at distance, and the NLOS environment is created by adding Gauss noise, Uniform noise and Poisson noise. The judgment of the LOS and NLOS is based on the variance. The distances from the anchor nodes to the unknown node are measured several times. If the variance of the obtained distance is greater than the set threshold, it is determined that the anchor node is in the NLOS environment at this time.

We evaluate the positioning accuracy of the least-squares method, the maximum likelihood method, the classical trilateration method, and the proposed method in the NLOS/LOS environment. For the other three methods, when the number of the anchor node is greater than or equal to 3, only the anchor nodes in the LOS environment are utilized to minimize the impact of the NLOS. When the number of the anchor node is smaller than 3, the anchor nodes in the NLOS environment will be utilized. However, for the proposed method, the selection of the anchor node will not be performed. Finally, we get the average positioning error and the average positioning time, as shown in Table 3. It can be seen from Table 3 that the error of positioning in the NLOS environment can be effectively reduced by using the proposed trilateration. Table 3 shows that the error of the proposed trilateration is 41.44% lower than that of the classical trilateration, 93.75% lower than that of the least-squares method, and 92% lower than that of the maximum likelihood method.

This is because the error of the trilateration increases for a single point. However, by utilizing K-Means clustering, the position of each centroid is influenced by the samples in the cluster. Therefore, the offset of the center of mass does not increase too much, and its error will correspondingly be less than that of the classical trilateration. However, the error decreases at the same time, sacrificing time. The positioning accuracy and positioning processing time of the classical trilateration and the proposed method in the NLOS environment are shown in Table 3. It can be seen that the positioning processing time of the optimal selection trilateration is related to the number of grouping groups and cluster clusters, and its running time is 1.86 fold that of the classical trilateration method.

## 5. Experiment

In the simulation evaluation, the positioning accuracy and the efficiency are simulated and analyzed. Although the noise is added to simulate the real environment, there are still some gaps with the real situation. We set experiments to verify the feasibility of the algorithm with a small number of the anchor nodes. In this section, the experimental setup is first described, and then the feasibility of the method is evaluated and compared with other localization methods.

### 5.1. Experiment Setting

**Figure 13:**
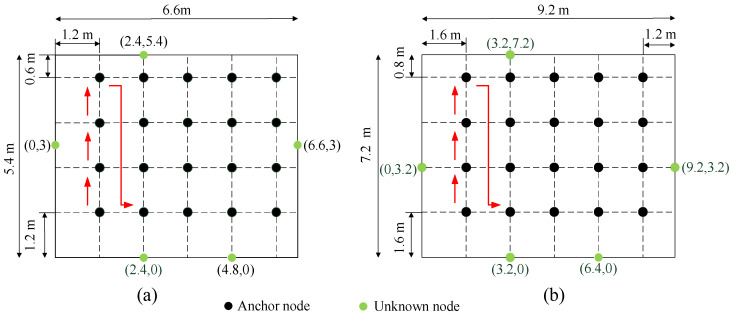
(**a**) Indoor localization field; (**b**) outdoor localization field and the hall. The black nodes represent the unknown node. The green nodes represent the anchor nodes. The red arrow represents the unknown node movement path.

There are five anchor nodes and an unknown node in this experiment, and the distance estimates of these nodes are obtained using RSSI. We deployed our localization system in three typical environments, i.e., indoor, outdoor and hall. The indoor localization range is a 5.4 m × 6.6 m field, as shown in Figure 13a. The outdoor and the hall localization range is a 7.2 m × 9.6 m field, as shown in Figure 13b.

In the above environment, the anchor node is fixed at specific positions, and the unknown node moves sequentially and sends ranging information to the anchor nodes to estimate the distance between the unknown node and the anchor nodes. For each position of the unknown node, ranging is performed 150 times, and the statistical calculations applied to these distances. After getting the average distances, the position of the unknown node can be obtained by using the positioning algorithms.

According to Formulas (1) and (2), the obtained RSSI data are transformed into distance information to obtain the data shown in Table 4. The horizontal header represents the label of the five anchor nodes. The vertical header represents the position where the unknown node has moved to. The data in the table represents the average distance measured by the anchor nodes when the unknown node moves to the specified position in a sequence in the three positioning environments.

### 5.2. Performance Evaluation

**Figure 14:**
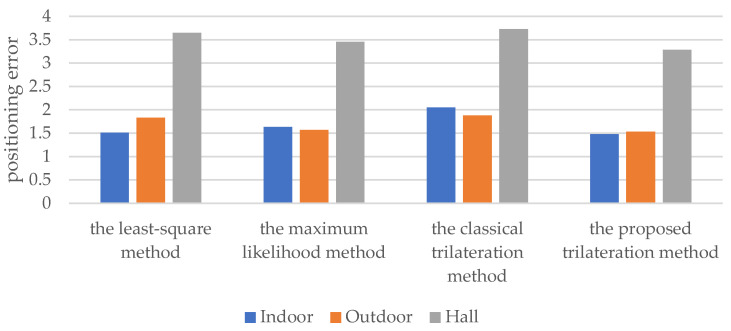
The positioning error of the four methods in different environments.

The localization accuracy and localization efficiency of the least-squares method, the maximum likelihood method, the classical trilateration localization method, and the proposed trilateration method were evaluated in the above localization environment. The average positioning errors of the various methods are shown in Figure 14 and Table 4.

It can be seen from Figure 14 that the positioning error of the improved trilateration method in indoor, outdoor and hall environments is lower than that of other methods.

In Table 5, the improved trilateration method can improve the positioning accuracy by 2.2%, 16.3% and 9.9% in indoor, outdoor and hall, respectively, compared with the least-squares method; compared with the maximum likelihood algorithm, the improved trilateration method can improve the positioning accuracy by 9.5%, 2.4% and 4.8% in indoor, outdoor and hall, respectively; compared with the classical trilateration positioning algorithm, the improved trilateration method can improve the positioning accuracy by 27.9%, 18.5% and 12.1% in indoor, outdoor and hall, respectively. This is mainly because the improved trilateration method can make full use of the information obtained by the anchor nodes and reduce the negative impact of uncertainty on the trilateration localization result and achieve higher localization accuracy.

### 5.3. Future Work

After the above discussion of the proposed trilateration method, we find that the positioning time of the proposed method is significantly higher than that of the other algorithms. This is because the fact that many repetitive calculations are performed in the process of localization, which is the biggest drawback of the proposed algorithm now. in our opinion, some advanced optimization algorithms can ameliorate this problem [3,49]. For example, using metaheuristics algorithms can find high-quality solutions in a short time. Using online learning can speed up the convergence of the algorithm to improve the efficiency of the algorithm. In the future work, advanced optimization algorithms [50,51] such as metaheuristics and genetic algorithms can be used to improve the algorithm.

## 6. Conclusions

To improve the performance and robustness of the trilateration positioning method, we present an improved trilateration method with anchor node combination and coordinate clustering strategy. Firstly, we combine the anchor nodes in the communication range of the unknown nodes into different trilateration groups. Then, for each group, we perform trilateration to gain the coordinate of the unknown node. Next, we cluster the coordinates with the K-Means clustering algorithm to determine the coordinate with high quality. Finally, we optimal select the cluster centroid as the positioning result. Comprehensive simulation results show that, compared with the classical trilateration algorithm, this method can effectively filter out the serious error caused by three circles not intersecting at one point, and can reduce the positioning error significantly, so that the positioning result is more accurate. In order to verify the effectiveness of the proposed method, we tested the proposed method in three environments, i.e., indoor, outdoor and hall.

There are still problems in this algorithm. In future research, this can be improved from the following aspects:

1. In order to obtain higher positioning accuracy, it is necessary to increase the number of estimated positions, which requires sacrificing a lot of time to calculate. Therefore, this method has a long positioning time and is not suitable for real-time positioning. Improving the positioning efficiency is one of the future research directions.
2. K-Means clustering uses random selection of initial cluster centers, which may require multiple iterations to reach a stable state in the iterative process. It is even possible that the final state arrived is not ideal. Therefore, in future research, different methods can be applied to determine more reasonable initial cluster centers.
3. In the research of this paper, all accessible anchor nodes are calculated, including many anchor nodes with large ranging errors. In future research, appropriate methods can be applied to screen the access anchor nodes, select anchor nodes with relatively small errors, and then use these anchor nodes for positioning.

## Figures and Tables

**Table 1:** The advantages and disadvantages of TOA, TDOA, AOA, and RSSI.

Measurement Method	Advantages	Disadvantages
TOA	TOA uses the round-trip time of messages to measure the distance between nodes, which has great ranging accuracy in terms of LOS environments.	TOA ranging technology usually requires pre-synchronization between nodes.
TDOA	TDOA uses the difference propagation rate between two different medium signals for distance measurement. Additionally, only one time of transmission is needed in TDOA.	Each node has to be equipped with a transceiver, which increases the cost of the node.
AOA	AOA uses the measured angle of arrival to achieve more accurate positioning.	More devices are required in AOA, which increases the cost and the size of the nodes.
RSSI	RSSI uses received signal strength to get the distance information. It is an excellent choice for low power and low complexity of signal processing.	The accuracy of RSSI mainly depends on the environment.

**Table 2:** Positioning error comparison of five methods.

Number of Anchor Nodes	The Least-Squares Positioning Error (m)	The Maximum Likelihood Positioning Error (m)	The Classical Trilateration Positioning Error (m)	The Proposed Method Positioning Error (m)
4	30.0796	29.5623	31.7795	30.0796
5	14.4165	26.2006	13.6152	14.4165
6	12.1168	41.3841	10.8887	12.1168
7	5.9310	37.3875	6.1745	5.9310
8	2.8003	19.2116	4.3791	2.8003
9	1.2607	64.4683	4.2965	1.2607

**Table 3:** The positioning error and positioning time comparison of the methods in the LOS/NLOS mixture environment.

Method	The Least-Squares Method	The Maximum Likelihood Method	The Classical Trilateration	The Proposed Method
Error (m)	32.5644	25.4902	2.2453	1.3148
Positioning time (s)	0.025	0.032	4.059	14.411

**Table 4:** The distances obtained from anchor nodes.

Distance (m)	Anchor 1			Anchor 2			Anchor 3			Anchor 4			Anchor 5		
	Hall	Indoor	Outdoor	Hall	Indoor	Outdoor	Hall	Indoor	Outdoor	Hall	Indoor	Outdoor	Hall	Indoor	Outdoor
**1**	3.2	3.11	3.04	6.03	4.24	4.33	9.33	7.3	6.99	6.89	5.72	5.9	3.71	3.44	3.33
**2**	4.92	3.74	4.04	6.33	5.44	5.05	8.73	6.43	6.72	5.64	4.81	4.36	2.9	2.52	2.49
**3**	6.03	5.59	5.22	7.78	6.23	5.73	13.26	7.01	6.68	3.83	3.52	3.34	3.47	2.6	2.52
**4**	8.85	6.56	5.98	8.99	6.43	6.23	9.33	6.59	6.96	2.81	2.66	2.6	4.99	3.4	3.41
**5**	2.73	2.3	2.37	4.56	3.49	3.41	7.32	5.9	5.75	6.86	5.79	5.31	4.82	4.67	4.17
**6**	4.18	3.63	3.4	5.48	4.62	3.93	7.26	5.91	5.47	4.62	4.41	4.16	4.39	3.99	3.66
**7**	6.34	4.44	4.78	7.17	5.36	4.89	7.41	5.4	5.5	3.01	3.24	3.02	4.88	3.24	3.72
**8**	8.44	6.83	5.97	7.35	6.82	6.21	8.39	6.22	6.93	2.01	1.75	2.57	5.87	4.63	3.4
**9**	3.4	3.21	2.91	2.78	2.22	2.37	5.75	5.74	4.43	6.84	5.9	5.61	6.22	6.32	5.13
**10**	4.7	3.88	3.88	4.82	3.61	3.18	5.97	4.08	4.14	5.73	4.92	4.22	6.22	5.14	4.94
**11**	6.44	5.66	4.9	6.07	4.48	4.39	5.72	4.82	4.15	4.39	3.68	3.45	6.31	5.71	5.14
**12**	8.06	6.43	6.26	7.66	6.33	5.68	6.62	3.81	4.4	2.88	2.74	2.58	7.35	5.73	5.44
**13**	4.76	4.66	3.9	2.28	2.26	2.09	4.42	4.22	3.43	8.36	7.14	6.1	8.8	6.58	6.57
**14**	5.83	4.89	4.58	4.18	4.08	3.22	3.95	3.51	3.09	6.34	6.29	5.13	8.9	8.9	6.04
**15**	7.11	5.89	5.67	4.86	4.5	4.45	4.38	3.48	3.05	4.86	5	4.34	8.39	7.47	5.96
**16**	8.73	8.12	6.81	7.74	6.73	5.7	5.47	4.25	3.61	4.46	4.21	3.58	8.58	7.18	6.28
**17**	6.18	5.47	4.7	2.74	3.32	2.42	2.93	3.65	2.93	8.44	4.87	6.77	11.28	9.42	7.77
**18**	7.17	6.58	5.6	4.03	3.49	3.38	2.31	3.04	2.25	7.58	7.69	5.92	9.83	8.76	7.34
**19**	7.4	7.76	6.34	6	5.4	4.4	3.24	3.36	1.99	6.6	6.92	5.24	9.46	7.59	7.48
**20**	9.28	9.01	7.3	7.26	7.01	5.74	4.37	4.08	3.05	6.31	6.22	4.71	11.22	7.4	7.73

**Table 5:** The positioning error of the four methods in different environments.

	Method	The Least-Squares Method	The Maximum Likelihood Method	The Classical Trilateration Method	The Proposed Trilateration Method
Environment					
**Indoor**		1.5118	1.6336	2.0504	1.4781
**Outdoor**		1.8308	1.5716	1.8819	1.5332
**Hall**		3.6500	3.4562	3.7300	3.2891

## Data Availability

Not applicable.
